# COASTAL: Combining Concolic and Fuzzing for Java (Competition Contribution)

**DOI:** 10.1007/978-3-030-45237-7_23

**Published:** 2020-03-13

**Authors:** Willem Visser, Jaco Geldenhuys

**Affiliations:** 8 Johannes Kepler University, Linz, Austria; 9 University of Birmingham, Birmingham, UK; Stellenbosch University, Stellenbosch, South Africa

## Abstract

COASTAL is a program analysis tool for Java programs. It combines concolic execution and fuzz testing in a framework with built-in concurrency, allowing the two approaches to cooperate naturally.

## 1 Verification Approach and Software Architecture

COASTAL analyses Java bytecode with an approach that combines concolic execution and fuzz testing in a unified framework. It uses the ASM bytecode manipulation library [2] to add code to compiled class files to monitor and interact with the system under test (SUT). The concurrent COASTAL components that carry out the analysis are shown in Figure 1:

- Multiple *divers* (for concolic analysis) execute the SUT with different concrete input values. A diver run is triggered when a vector of concrete input values is added to the diver input queue *d_in*. As a diver executes, the instrumented code mirrors the state of the program with symbolic values. At the end of the run, the symbolic path condition that corresponds to the execution is enqueued in the diver output queue *d_out*.
- Multiple *surfers* (for fuzzing analysis) also execute the SUT with concrete input values. A surfer run is triggered when a vector of concrete input values is added to the surfer input queue *s_in*. As a surfer executes, lighter instrumentation records the "shape" of the execution path, and at the end of the run, this information is enqueued in the surfer output queue *s_out*.
- One or more strategies remove and process the information that appears on the diver and surfer output queues. For example, a strategy may remove a path condition, negate one or more constraints, invoke an SMT solver to find input values that will explore the modified path, and enqueue them on *d_in* or *s_in* or both. Instrumentation injects the input values into the SUT.
- To share information between components, discovered execution paths are stored in a shared execution tree known as the *pathtree*. The pathtree keeps track of which sub-trees have been fully explored. The pathtree data structure allows for efficient concurrent updates.

* Jury member

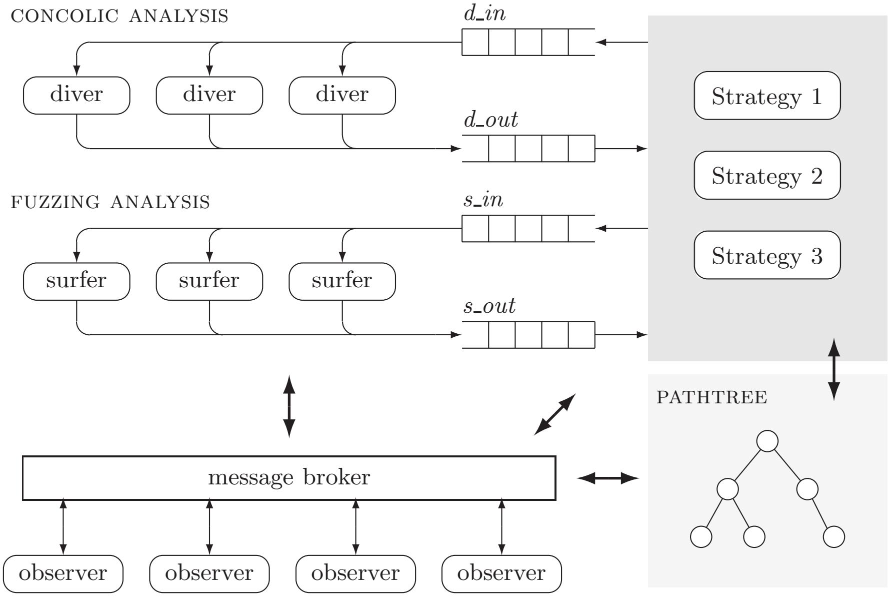

**Fig. 1.** COASTAL architecture

– Divers, surfers, strategies, and the pathtree signal their actions via a publish-subscribe system. When events are published to the message broker, one or more observers are notified. The observers may, in turn, emit messages that direct the operation of COASTAL.

### 1.1 Strategies

As an example, a *depth-first strategy* is a simple configuration of COASTAL where the strategy employs only a single diver. The diver produces one path condition that is processed by the strategy by negating the last (deepest) constraint, and sending it to an SMT solver, which produces new input values (if any) that will explore the modified path. If a modified path condition is unsatisfiable, the last constraint is discarded and the process repeats. All path conditions are added to the pathtree as they are discovered. At the end of the analysis, the pathtree contains a summary of the execution tree of the SUT.

Other strategies include *breadth-first* and *random* exploration. Like depth-first exploration, these strategies use only one diver and explore one path condition at a time. On the other hand, a *generational strategy* negates all the constraints of a path condition, one by one, and produces many potential input values. In this case, multiple divers can be used concurrently. Users can also deploy multiple strategies at the same time.

**Fuzzing strategies.** The user can employ surfers to perform straightforward fuzz testing (in the style of AFL [1,5,6]). Surfers use very little instrumentation.

Unlike the divers — that instrument every bytecode instruction — only the outcomes of branching points are recorded. The "path condition" produced by a surfer is therefore a series of (mostly binary) choices that can be added to the pathtree; it lacks any details about the reason for the choice (for example, instead of "$x > 5$" it may simply record "*false*"), but the shape of the path is preserved. Multiple divers and multiple surfers are deployed concurrently and operate interactively.

**Hybrid strategies.** More advanced strategies can combine concolic and fuzzing analysis to exploit the strengths of both approaches: surfers (fuzzing) can rapidly explore new territory of the execution space, while divers (concolic) can investigate hard-to-reach corners. Such hybrid strategies enqueue (semi-)random inputs on *s_in* and the results contribute to a "skeletal" pathtree. Since surfers produce results at a high rate, the easy-to-explore parts of the execution space are more quickly saturated. Unexplored regions of the pathtree are passed to the divers, and their results, in turn, open up new regions for the surfers to explore.

### 1.2 Observers and Models

COASTAL was designed with extensibility in mind. One example is the use of *observers*. Any component is allowed to subscribe to the various message streams, and can interact with the system by publishing messages of their own, or by making direct calls to the public COASTAL API. Examples of observer tasks include:

- monitor assertions and halt COASTAL when they are violated,
- record instruction, line, and condition coverage,
- enforce assumptions and prune undesired execution paths,
- gather information and display progress in a GUI.

In theory, strategies themselves could be implemented as observers. But since they are central to the operation of COASTAL, they are given special treatment.

Users can replace system- or user-level libraries by more appropriate *models*, either as a whole or on a method-by-method basis. For example, a complex library implementation of `String.substring()` can be replaced with a simpler, more efficient model that produces the same result and the same symbolic constraints.

## 2 Strengths and weaknesses

The tool's strength lies in the combination of concolic and fuzzing analysis, but COASTAL is still under development and a "deep" bug (now fixed) prevented the use of fuzzing. Participation in SV-COMP [3] was invaluable in this regard: Several bugs and missing functionality were revealed and corrected.

**Results.** COASTAL does not output any incorrect answers, but produces an *unknown* result in 19% of cases. This is shown in column "Count" below.

| Answer | Count | | Immediate | |
|---|---|---|---|---|
| *true* | 135 | 32.45% | 121 | 89.63% |
| *false* | 202 | 48.57% | 134 | 66.34% |
| *unknown* | 79 | 18.99% | 27 | 34.18% |
| All | 416 | 100% | 282 | 67.79% |

For many cases, the answer is produced instantaneously (column "Immediate"). In the case of *unknown* answers, this indicates that COASTAL aborted its analysis because of an as-yet unsupported feature such as symbolic array sizes. For the $79 - 27 = 52$ non-immediate *unknown* answers, COASTAL timed out because of large search spaces.

The longest-running *true* answer required 2 diver runs, each taking 20.48sec (`printtokens_eqchk.yml`), whereas the longest-running *false* answer required 141 diver runs, each taking 0.54sec (`spec1-5_product1.yml`). This highlights a fundamental weakness of the tool: a long-running SUT takes longer to analyse. A generational strategy where multiple divers execute concurrently can ameliorate this problem, but on average does not find errors as quickly as the breadth-first strategy. This points to the need to refine the generational strategy to prioritize shallow unexplored paths.

## 3 Tool setup

**Download.** http://doi.org/10.5281/zenodo.3679243 [7]

**Configuration.** COASTAL is configured to use a breath-first search strategy and a single diver. Z3 [4] is set as the constraint solver. (It is the only external tool required to run COASTAL and a Linux executable version is included in the download above.) Path conditions are limited to 800 conjuncts, and a time limit of 240 second is set. Symbolic strings are limited to 25 characters. Custom models are used for some Java classes: `Character`, `String`, `StringBuilder`, `Pattern`, `Matcher`, `Scanner`. COASTAL competed in the `JavaOverall` category.

**Installation.** The download above is self-contained. The COASTAL project at https://github.com/DeepseaPlatform/coastal/ includes shell scripts to package and run COASTAL for SV-COMP in the `extra/svcomp` subdirectory. The scripts needs an external copy of the Z3 solver to be available.

## 4 Software Project

COASTAL is developed by the authors at Stellenbosch University, South Africa. It is available at https://github.com/DeepseaPlatform/coastal/ and is distributed under the GNU Lesser General Public License version 3.
